# Therapy-related acute myeloid leukemia with 24-month latency after CD19 CAR-T cell therapy in relapsed/refractory diffuse large B-cell lymphoma: a case report

**DOI:** 10.3389/fmed.2026.1876141

**Published:** 2026-08-14

**Authors:** Manqi Su, Chentong Huang, Zhanna Zhang, Xiaoxia Wang, Panruo Jiang, Yiran Chen, Gongqiang Wu

**Affiliations:** 1 Department of Hematology, Dongyang People's Hospital, Dongyang Hospital Affiliated to WenZhou Medical University, Dongyang, Zhejiang, China; 2 Hangzhou City University, Hangzhou, Zhejiang, China

**Keywords:** chimeric antigen receptor (CAR) T cells, refractory/relapsed B-cell lymphoma, second cancer, therapy-related acute myeloid leukemia (t-AML), therapy-related myelodysplastic syndrome (t-MDS)

## Abstract

**Background:**

Therapy-related myeloid neoplasms (t-MNs) have emerged as a serious late complication of CD19-directed chimeric antigen receptor (CAR) T-cell therapy. While large-scale studies have characterized the epidemiology of t-MNs, the mechanisms underlying their development remain incompletely understood, and cases with extended latency are rarely documented. We report a case of therapy-related acute myeloid leukemia (t-AML) occurring 24 months after initial CD19 CAR-T cell therapy, with longitudinal genomic monitoring demonstrating evolution from wild-type TP53 to biallelic TP53 loss.

**Case presentation:**

A 65-year-old male was diagnosed with germinal center B-cell (GCB)-type diffuse large B-cell lymphoma (DLBCL) in February 2019. After first-line R-CHOP chemotherapy and subsequent relapse, he received two courses of CD19 CAR-T cell therapy (June 2020 and February 2021) with sintilimab maintenance. Twenty-four months after the first CAR-T infusion, he presented with pancytopenia. Bone marrow examination revealed increased primitive and immature mononuclear cells, with flow cytometry confirming 9.49% abnormal myeloid blasts (CD34+, CD117+, MPO+) consistent with AML-M5b. Cytogenetic analysis demonstrated a complex karyotype: 44-45, XY, del(5)(q14q33),−16, der(17;18)(q10;q10),-19, add(21q). Next-generation sequencing identified biallelic TP53 loss (copy number loss and p.P278A point mutation in exon 8). Notably, NGS at initial DLBCL diagnosis had shown wild-type TP53. The patient received azacitidine plus venetoclax, achieving partial remission, followed by child-to-parent haploidentical allogeneic hematopoietic stem cell transplantation. DLBCL remained in remission throughout AML-directed therapy. The patient ultimately died from COVID-19 pneumonia in December 2022.

**Conclusion:**

This case illustrates a rare but serious late complication of CD19 CAR-T cell therapy, characterized by an exceptionally long 24-month latency and documented evolution from wild-type TP53 to biallelic loss. While causality cannot be established from a single case, these findings are consistent with the hypothesis that prolonged genotoxic stress and inflammatory cytokine exposure may promote clonal selection of TP53-mutated hematopoietic cells. This report underscores the necessity of extended hematologic surveillance beyond the first 12 months post-CAR-T infusion, baseline CHIP screening prior to therapy, and systematic longitudinal genomic profiling in future cases to validate these observations.

## Introduction

Therapy-related myeloid neoplasms (t-MNs), including therapy-related myelodysplastic syndrome (t-MDS) and therapy-related acute myeloid leukemia (t-AML), have emerged as a significant late complication of CD19-directed chimeric antigen receptor (CAR) T-cell therapy. While CAR-T cell therapy has revolutionized the treatment of relapsed/refractory (R/R) diffuse large B-cell lymphoma (DLBCL) with objective response rates of 75.9–83%, the long-term safety profile continues to evolve (1, 2). However, these epidemiological data are derived from relatively short follow-up periods (median 6.7–25 months), and the mechanistic underpinnings of t-MN development remain incompletely characterized.

The pathogenesis of t-MN is multifactorial, involving prior cytotoxic chemotherapy, radiotherapy, and potentially the inflammatory microenvironment created by CAR-T cell therapy. Preclinical studies have demonstrated that inflammatory cytokines, including interleukin-6 (IL-6), tumor necrosis factor-alpha (TNF-alpha), and interferon-gamma (IFN-gamma), can impose genotoxic stress on hematopoietic stem and progenitor cells (HSPCs), promoting the selective expansion of TP53-mutated clones (3). In patients with TP53-mutated AML, single-cell multi-omics analysis has revealed that chronic inflammation drives clonal evolution and malignant transformation (4). Nevertheless, clinical evidence validating this mechanistic model remains limited, especially longitudinal genomic profiles covering the full disease course from the initial diagnosis of lymphoma to the development of t-MN.

Diffuse large B-cell lymphoma (DLBCL), the most common non-Hodgkin lymphoma, is initially treated with R-CHOP, but 30–40% of patients relapse or become refractory (5, 6). For these patients, CD19 CAR-T therapy offers substantial efficacy, yet the cumulative genotoxic exposure from prior chemotherapy, coupled with CAR-T-associated inflammatory cytokine release, may create a permissive environment for clonal hematopoiesis and subsequent myeloid neoplasia. The immunomodulatory agent lenalidomide, used in this patient's maintenance and salvage regimens, has additionally been associated with secondary malignancies in other contexts, although its specific contribution to t-MN in DLBCL remains undefined.

Here, we report a case of t-AML diagnosed 24 months after the first CD19 CAR-T cell infusion in a patient with R/R DLBCL. This case is notable for several distinctive features: an exceptionally long latency period, documented evolution from wild-type TP53 at DLBCL diagnosis to biallelic TP53 loss at t-AML diagnosis, sustained DLBCL remission during AML-directed therapy, and a complex treatment history involving two courses of CAR-T therapy, lenalidomide exposure, and PD-1 inhibitor maintenance. This report aims to present detailed longitudinal genomic characterization of clonal evolution and to explore the potential interplay between prolonged genotoxic stress and CAR-T-associated inflammatory signaling in driving TP53-mediated malignant transformation.

## Case report

**Figure 1:**
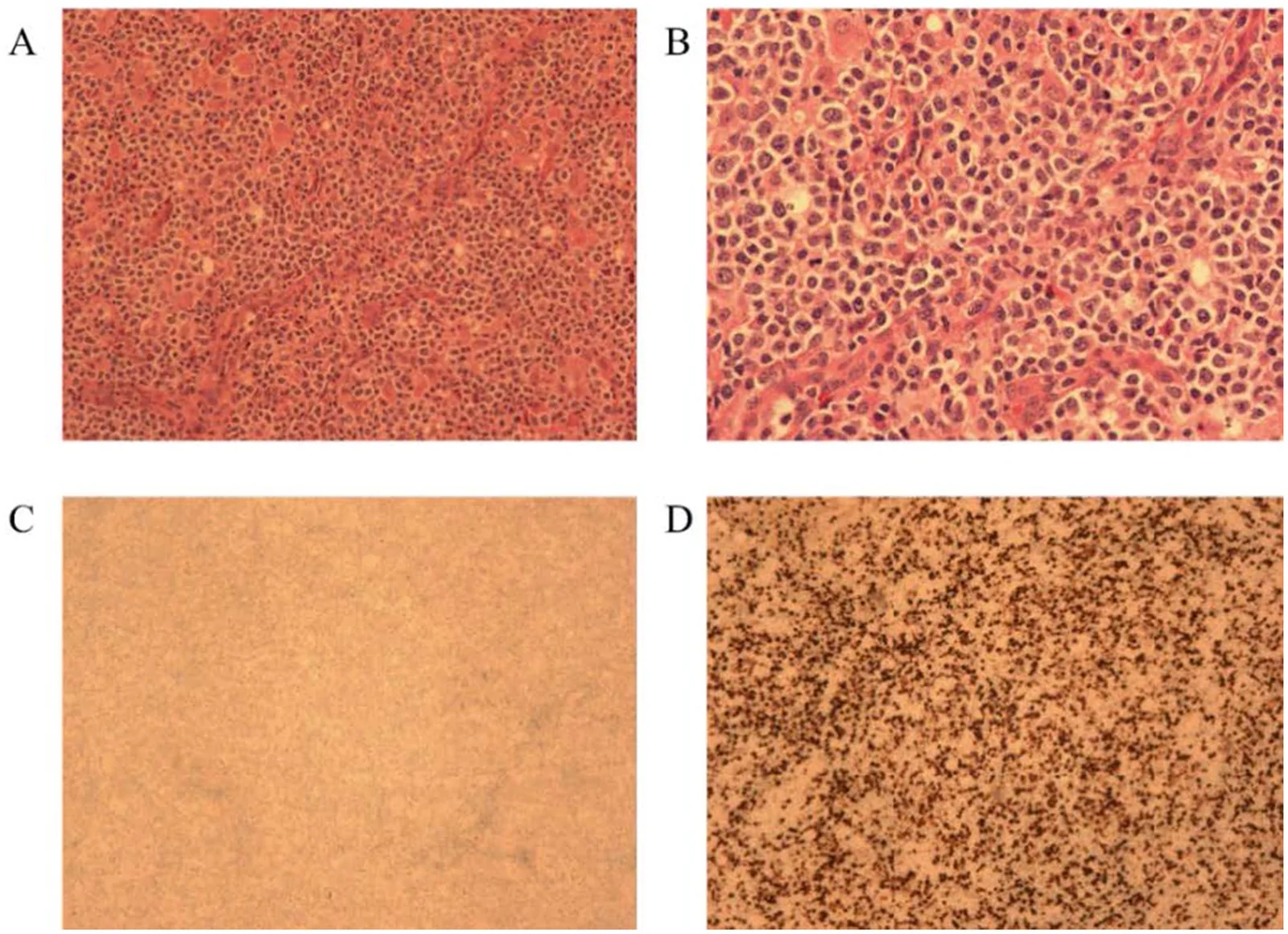
Pathological and immunohistochemical features of cervical lymph node biopsy. Hematoxylin-eosin (HE) staining [**(A)**: 100×, **(B)**: 200×] demonstrates prominent abnormal lymphoid proliferation. Immunohistochemistry reveals diffuse positivity for B-cell markers (CD20, CD79a, PAX5, BCL-2, BCL-6) with negativity for MUM1, CD10, CD23, CD21 and Cyclin D1, plus scattered T-cell positivity (CD3, CD5, CD43). EBER shows focal positivity, and the Ki-67 proliferation index is ~70%. **(C)** is CD10 immunohistochemistry (negative), and **(D)** is Ki-67 immunohistochemistry (high proliferation rate).

The patient was a 65-year-old male. In February 2019, he was admitted to DongYang People's Hospital because of multiple neck masses for more than half a month. His routine blood test results were as follows: a white blood cell (WBC) count of 2.9x10^9/*L*^; a red blood cell (RBC) count of 2.98x10^12/*L*^; a hemoglobin (Hb) level of 86g/L, and a platelet (PLT) count of 96x10^9/*L*^. Biochemical tests were normal. Cervical lymph node biopsy revealed diffuse proliferation of large atypical lymphoid cells. Pathological assessment demonstrated effacement of normal architecture by a monotonous population of large lymphoid cells with irregular nuclei and prominent nucleoli, consistent with diffuse large B-cell lymphoma. Immunohistochemical staining revealed scattered positivity for CD3, CD5 and CD43; EBER was focally positive, and the Ki-67 proliferation index reached approximately 70%. Bone marrow aspirate showed no atypical lymphoid infiltration, with a normal karyotype (Figure 1). The patient was diagnosed with Germinal Center B-cell type diffuse large B-cell lymphoma (GCB-type DLBCL). After diagnosis, the patient received 4 cycles of R-CHOP chemotherapy and achieved complete remission. Complications from chemotherapy included bone marrow suppression and pulmonary infection, managed with recombinant human granulocyte colony-stimulating factor (rhG-CSF) and antimicrobial therapy. A PET/CT scan of the patient in September 2019 indicated malignant intracranial lesions accompanied by peripheral edema. Rituximab combined with high-dose methotrexate and temozolomide was administered via intrathecal injection, and oral lenalidomide maintenance therapy was given.

In April 2020, a follow-up examination at the local hospital revealed recurrence through PET/CT imaging. The patient was subsequently enrolled in a clinical study conducted by Shanghai Yake Biotechnology Company, which assessed the safety and efficacy of humanized anti-CD19 CAR-T cell therapy for the treatment of B-cell acute lymphoblastic leukemia and B-cell non-Hodgkin's lymphoma. Following the successful ex vivo expansion of CAR-T cells, the patient underwent lymphodepletion with a fludarabine and cyclophosphamide regimen. Subsequently, the patient received CAR-T cell infusion on June 5, 2020, and did not exhibit any significant pyrexia or other clinical signs of cytokine release syndrome (CRS) post-infusion. A follow-up PET/CT scan conducted on July 7, 2020, indicated a marked reduction in tumor size compared to previous assessments. On September 9, 2020, three months after CAR-T cell infusion, a PET/CT scan revealed that the patient achieved a partial response to the treatment.

**Figure 2:**
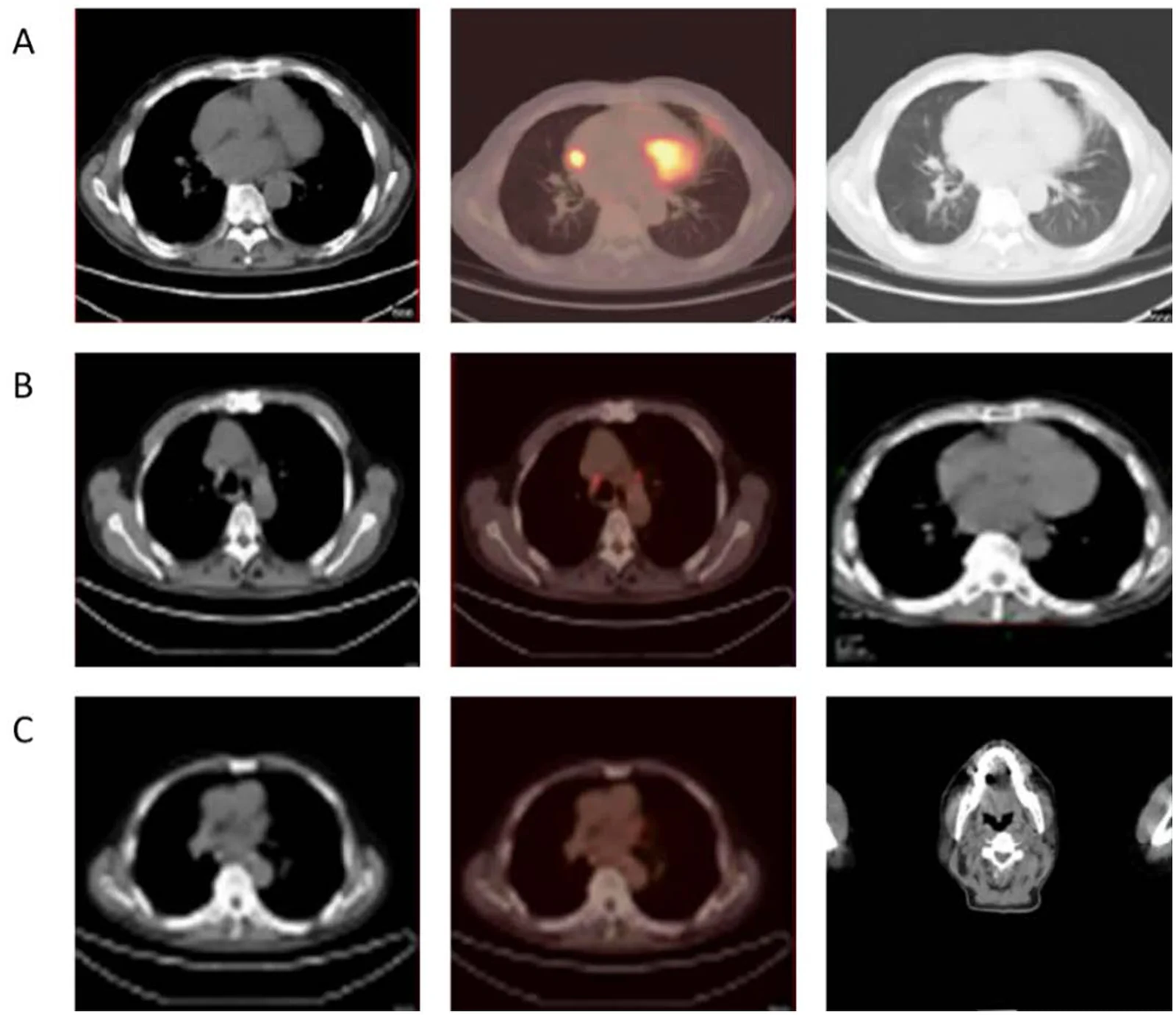
**(A)** Shows PET/CT scans acquired on May 8, 2020, confirming disease recurrence. **(B)** Corresponds to PET/CT imaging dated July 7, 2020, demonstrating marked tumor shrinkage vs. prior scans. **(C)** Illustrates PET/CT results from December 7, 2020; elevated FDG uptake is detected in multiple lymph nodes of the left neck, left supraclavicular fossa and left axilla, consistent with recurrent disease.

On December 17, 2020, PET/CT revealed increased FDG metabolism in the left neck, left supraclavicular fossa, and left axillary multiple lymph nodes, indicating disease recurrence. The “rituximab + lenalidomide + Zevalin” therapeutic regimen was used, and then CD19 CAR-T cell therapy was administered again at a local hospital in February 2021, followed by 3 rounds of sintilimab treatment. After complete remission, sintilimab maintenance therapy was administered 18 times. The results of the previous PET/CT scans are shown in Figure 2.

**Figure 3:**
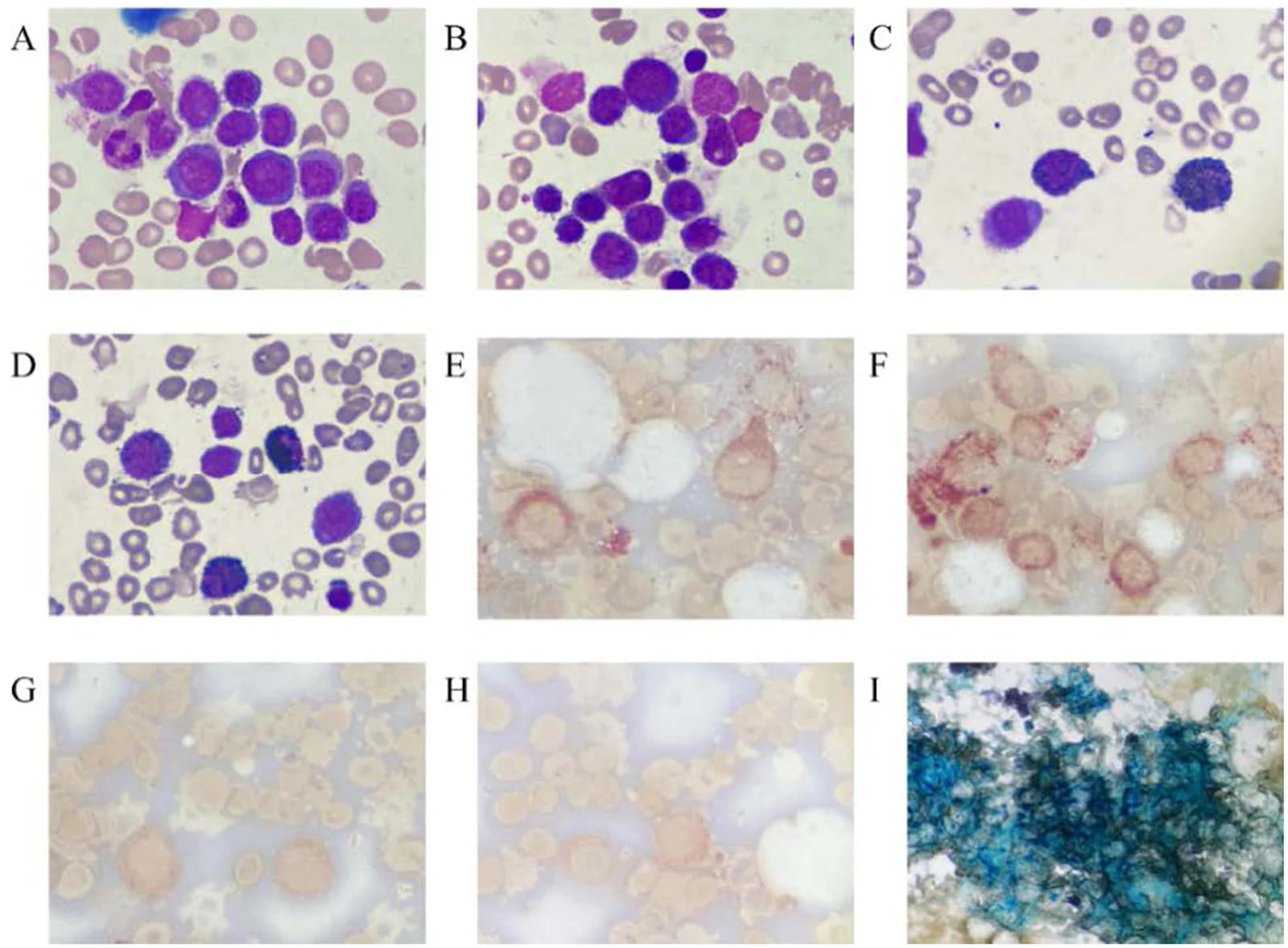
Bone marrow cytology examination: The ratio of primitive to young mononuclear cells was significantly elevated. Bone marrow biopsy suggested an increased proportion of primitive and immature cells in the marrow cavity. **(A, B)** Depict the morphology of bone marrow cells, **(C, D)** Show myeloperoxidase (MPO), **(E, F)** Represent non-specific esterase (NAE), **(G, H)** Depict NAE and Naphthol AS-D chloroacetate esterase (NAF) staining, and **(I)** Shows iron staining.

**Figure 4:**
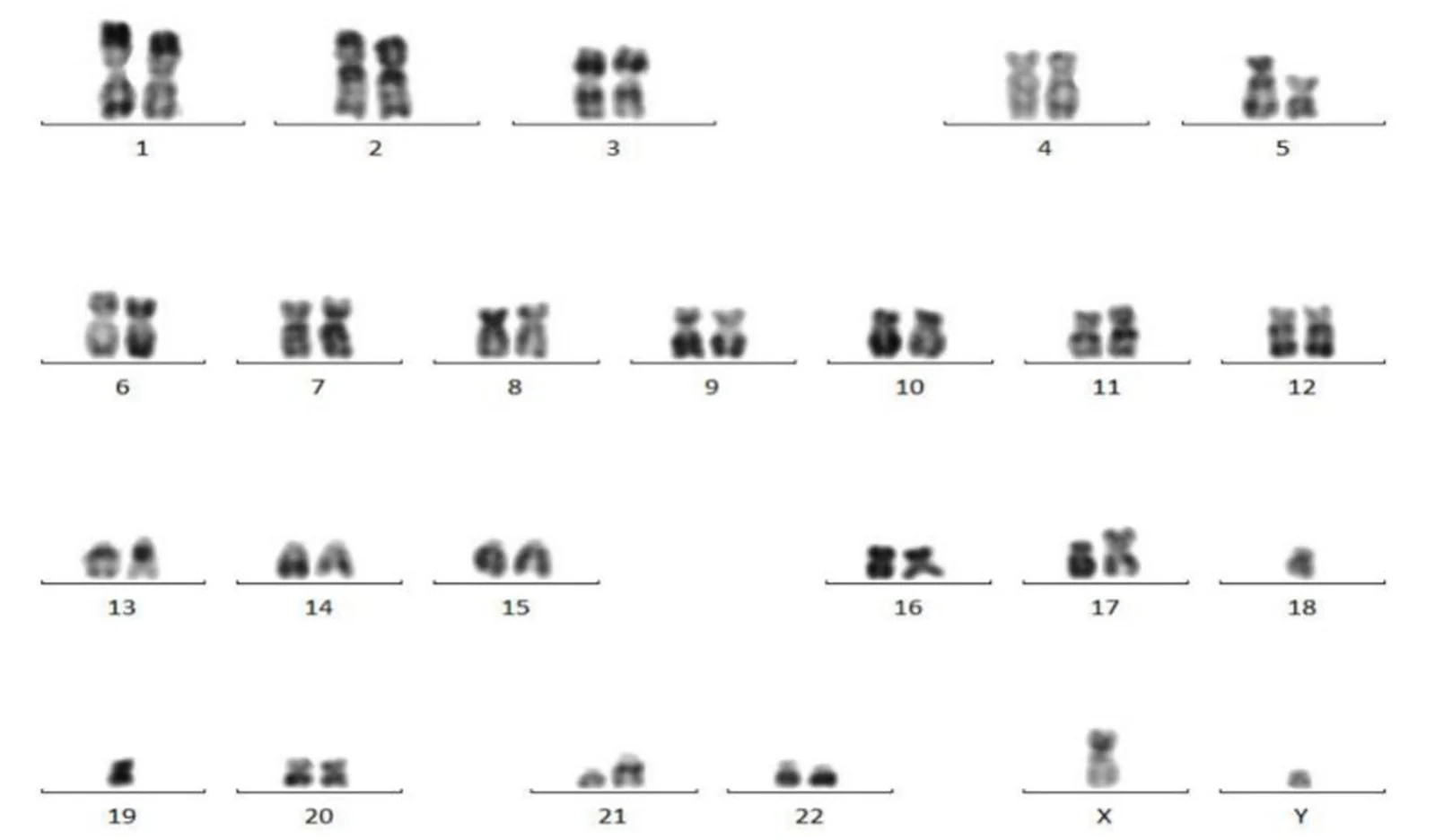
Karyotype: 44-45, XY, del (5) (q14q33),−16, der (17;18)(q10; q10),-19, add (21q) [cp15]/46, XY [5]. Conclusion: Clonal abnormalities del(5q),−16, der (17;18),−19, and add(21q) are observed.

At the end of June 2022, the patient was brought to DongYang People's Hospital due to pancytopenia. The complete blood count (CBC) showed the following results: WBC of 2.57 x 10^9/*L*^; RBC of 1.98 x 10^12/*L*^; Hb of 68g/L; and PLT of 35 x 10^9/*L*^. A bone marrow examination, including a smear and biopsy, indicated a significant increase in the proportion of primitive and immature mononuclear cells (Figure 3). Flow cytometry immunophenotyping revealed that myeloid blasts accounted for 15% of nucleated cells, and immature mononuclear cells accounted for 38%, leading to a diagnosis of AML-M5b. Cytogenetic analysis revealed clonal abnormalities including del(5q),−16, der (17;18),−19, and add(21q) (Figure 4). Next-generation sequencing (NGS) results indicated a copy number loss of TP53 and a point mutation in exon 8, p.P278A, consistent with biallelic loss of TP53, suggesting a poor prognosis for the patient. After the patient initially declined chemotherapy and was readmitted in July 2022 for reassessment, repeat bone marrow examination showed 11% primitive and immature mononuclear cells, with flow cytometry confirming 9.49% abnormal myeloid blasts (CD34+, CD117+, MPO+) and no evidence of abnormal B lymphocytes. The CBC showed a WBC of 0.93 x 10^9/*L*^, RBC of 2.02 x 10^12/*L*^, Hb of 69 g/L, and PLT of 40 x 10^9/*L*^. Peripheral blood smear showed marked leukopenia with neutropenia; no circulating blasts were identified. The patient was then treated with a combination therapy of azacitidine and the Bcl-2 inhibitor venetoclax. Examinations conducted in August and September 2022 indicated a partial remission (PR) of AML, with the lymphoma in a state of ongoing remission. Allogeneic hematopoietic stem cell transplantation (from child to parent) was performed in October 2022. Post-transplantion, there was no significant graft vs. host disease (GVHD), and the patient received treatment with cyclosporine and steroids for GVHD prophylaxis. In December 2022, the patient suffered from COVID-19 infection with progressive pulmonary infiltrates, ultimately leading to respiratory failure and death.

## Diagnostic rationale and differential diagnosis

**Figure 5:**
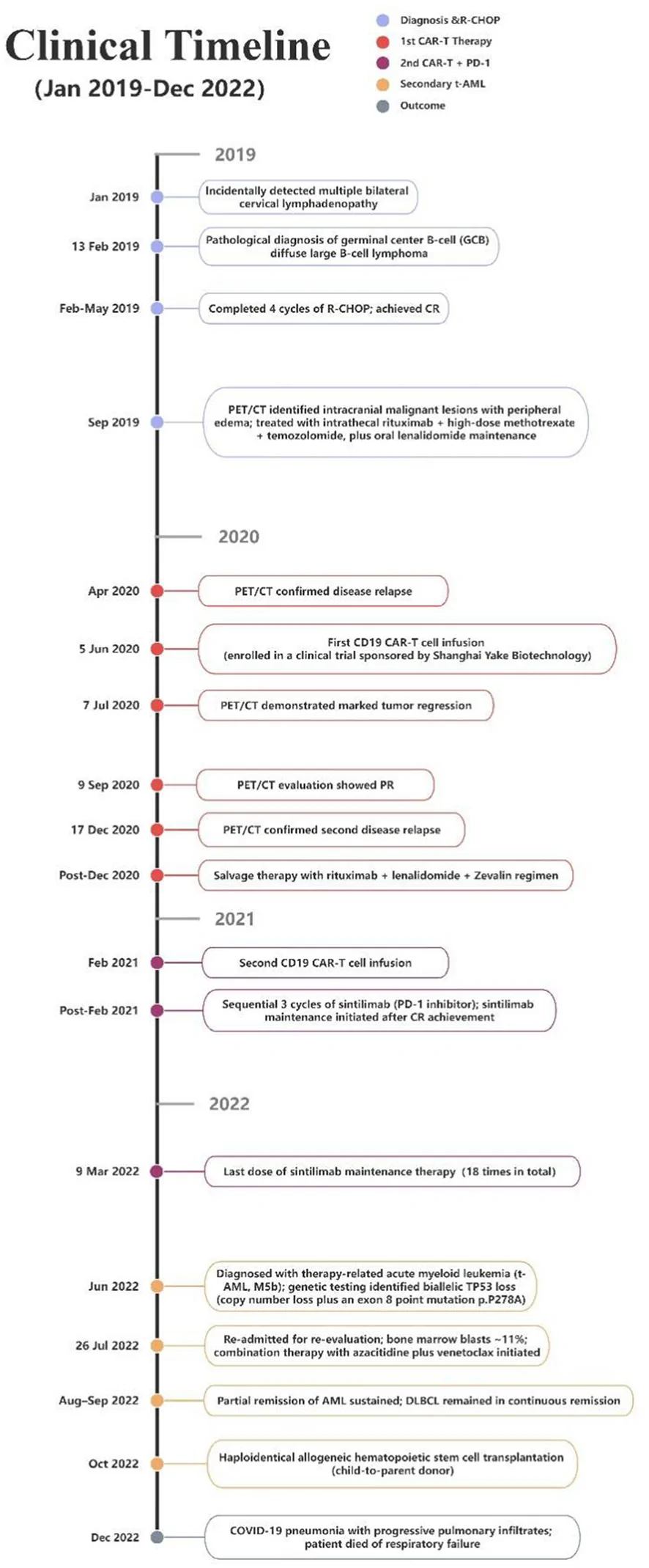
Clinical timeline of the patient's disease course from initial DLBCL diagnosis to death. The patient received first-line R-CHOP chemotherapy (blue), followed by two courses of CD19 CAR-T cell therapy (red and purple) with sintilimab maintenance. Therapy-related acute myeloid leukemia (t-AML, M5b) was diagnosed 24 months after the first CAR-T infusion (orange). The patient ultimately underwent haploidentical allogeneic hematopoietic stem cell transplantation but died from COVID-19 pneumonia in December 2022 (gray).

The diagnosis of therapy-related acute myeloid leukemia (t-AML) was established based on a combination of morphologic, immunophenotypic, cytogenetic, and molecular findings. Several lines of evidence supported this diagnosis and distinguished it from a relapse of the prior DLBCL or marrow infiltration by lymphoma. First, flow cytometry identified a myeloid blast population (CD34+, CD117+, MPO+) with no detectable aberrant B lymphocytes, effectively excluding lymphoma involvement. Second, the complex karyotype and biallelic TP53 loss identified at t-AML diagnosis were absent at the time of initial DLBCL diagnosis, indicating a clonally unrelated secondary process rather than evolution of the original lymphoma clone. Third, the patient maintained clinical and radiological remission of DLBCL throughout AML-directed therapy, further supporting the independent origin of the two malignancies. The clinical timeline of this patient is summarized in Figure 5.

## Discussion

### Epidemiology of t-MN following CAR-T cell therapy

The second pattern results in therapy-related myeloid neoplasms (t-MNs), primarily therapy-related myelodysplastic syndrome (t-MDS) and therapy-related acute myeloid leukemia (t-AML) (7). Therapy-related myeloid neoplasms (t-MNs), primarily t-MDS and t-AML, represent a recognized late complication of CD19 CAR-T therapy, with cumulative incidence estimates ranging from 2.3% to 4.5% in large cohorts (8, 9). These data establish t-MN as a clinically significant late complication, although the absolute risk remains relatively low. Importantly, t-AML is distinctly rare compared with t-MDS, and previously reported cases occurred within 2–3 months of infusion (7, 10, 11). Our case is unique in its 24-month latency from the first CAR-T infusion, substantially longer than previously reported intervals. This extended interval raises critical questions about the duration of risk and the necessity for prolonged hematologic surveillance beyond the typical follow-up periods of clinical trials.

### Distinctive features of the present case

Several features of this case warrant particular attention. First, the 24-month latency from first CAR-T infusion to t-AML diagnosis is substantially longer than previously reported. While the median interval between CAR-T infusion and secondary myeloid neoplasm diagnosis was 6.7 months in the ClonHema cohort (range 1.3–40.6 months) (9), the three prior t-AML cases occurred within 2–3 months, suggesting a narrow early risk window. Our case demonstrates that t-AML can present as a late complication, challenging the assumption that the risk is confined to the immediate post-infusion period.

Second, the documented evolution from wild-type TP53 at DLBCL diagnosis to biallelic TP53 loss (copy number loss plus p.P278A point mutation) at t-AML diagnosis provides direct clinical evidence for a dynamic clonal selection process. In the French multicenter study, Gazeau et al. reported that 85.7% of t-MN cases harbored pre-existing mutations, including TP53 variants, suggesting that clonal hematopoiesis of indeterminate potential (CHIP) may predispose to t-MN (8). However, that study did not demonstrate the temporal evolution from wild-type to mutated status. Our longitudinal genomic data support the hypothesis that prolonged genotoxic stress—stemming from prior chemotherapy, CAR-T-related inflammation, and potentially lenalidomide exposure—creates selective pressure favoring the expansion of TP53-deficient HSPCs.

Third, the patient maintained DLBCL remission throughout AML-directed therapy. This observation suggests that the t-AML and DLBCL represent independent clonal processes rather than treatment failure of the lymphoma. The complex karyotype with del(5q),−16, der(17;18),−19, and add(21q) is characteristic of therapy-related myeloid neoplasms and further supports a treatment-related etiology distinct from the lymphoma clone.

### Mechanistic considerations

The pathogenesis of t-AML following CAR-T therapy likely involves the interplay of multiple factors. The patient in this case had substantial prior genotoxic exposure, including four cycles of R-CHOP chemotherapy (cyclophosphamide, doxorubicin), high-dose methotrexate for central nervous system prophylaxis, and two courses of fludarabine-cyclophosphamide lymphodepletion. These agents are well-established causes of therapy-related myeloid neoplasms through DNA damage, marrow stem cell depletion, and creation of a proinflammatory microenvironment (12).

Beyond direct DNA damage, the inflammatory environment following CAR-T cell therapy may play a critical role in clonal selection. CD19 CAR-T cell infusion is associated with cytokine release syndrome (CRS) and immune effector cell-associated neurotoxicity syndrome (ICANS), characterized by elevated levels of IL-6, IL-10, GM-CSF (13, 14). These inflammatory cytokines can induce DNA damage and oxidative stress in HSPCs. In the context of TP53 deficiency, which impairs DNA repair and apoptotic responses, cytokine-induced genotoxic stress may preferentially promote the survival and proliferation of mutated clones while suppressing wild-type hematopoiesis. This mechanism is supported by elegant single-cell multi-omics studies, which demonstrate that chronic inflammation drives TP53-mutant clonal evolution and leukemic transformation (3, 4).

The role of lenalidomide deserves specific consideration. The patient received lenalidomide as maintenance therapy from September 2019 to April 2020 (approximately 7 months) and again as part of the rituximab-lenalidomide-Zevalin salvage regimen in December 2020, totaling approximately 9 months of exposure. Lenalidomide, an immunomodulatory imide drug (IMiD), has been associated with an increased risk of secondary malignancies, including myeloid neoplasms, in multiple myeloma patients receiving long-term maintenance (15). While the mechanism is not fully elucidated, IMiDs may promote genomic instability through cereblon-dependent effects on DNA damage responses and altered DNA repair pathways. In the context of this patient, lenalidomide exposure may have contributed to the genotoxic milieu, potentially synergizing with prior chemotherapy and CAR-T-related inflammation. However, given the absence of definitive evidence establishing lenalidomide as an independent risk factor for t-MN in DLBCL, and the confounding effects of multiple prior therapies, we cannot attribute causality.

### Clinical implications

This case carries several important clinical implications. First, the 24-month latency underscores the necessity for extended hematologic monitoring in CAR-T recipients. Current follow-up protocols typically focus on the first 12 months post-infusion; our case suggests that cytopenias developing beyond this window should prompt thorough evaluation, including bone marrow examination with cytogenetic and molecular studies. Second, the documented TP53 evolution supports the rationale for baseline CHIP screening prior to CAR-T therapy, as recommended by recent consensus guidelines. Identifying pre-existing TP53 mutations or other CHIP variants could inform risk stratification and monitoring intensity. Third, the sustained DLBCL remission during AML therapy suggests that t-MN and lymphoma progression are independent processes, and the development of t-MN should not be interpreted as CAR-T failure.

### Strengths and limitations

#### Strengths

This case provides longitudinal genomic monitoring spanning from initial DLBCL diagnosis through t-AML evolution, a feature rarely available in the literature. The comprehensive diagnostic workup, including morphology, flow cytometry, cytogenetics, and next-generation sequencing, ensures robust diagnostic certainty. The detailed treatment history enables assessment of cumulative genotoxic exposure, and the clear documentation of DLBCL remission during AML therapy supports the independent clonal nature of the two malignancies.

#### Limitations

As a single-case report, this study is inherently limited in generalizability. No pre-CAR-T screening for clonal hematopoiesis of indeterminate potential (CHIP) was performed, as this was not standard practice in 2020; thus, we cannot definitively exclude the presence of a low-burden pre-existing clone. The follow-up was terminated by the patient's death from COVID-19 pneumonia, preventing assessment of long-term t-AML trajectory and tran**s**plant outcomes. Additionally, we cannot definitively attribute t-AML to CAR-T therapy vs. prior chemotherapy (R-CHOP, high-dose methotrexate) or lenalidomide maintenance, as these exposures represent confounding factors. Cytokine levels during CAR-T therapy were not prospectively measured, limiting direct mechanistic evidence for the inflammation-driven clonal selection hypothesis. Finally, the NGS panel used at DLBCL diagnosis (139-gene lymphoma-focused panel) differed from that used at t-AML diagnosis (523-gene myeloid malignancy panel), which may have affected the sensitivity for detecting low-burden CHIP clones. Additionally, the absence of serial bone marrow sampling between DLBCL diagnosis and t-AML onset precludes precise determination of when the TP53 mutation emerged, representing a key limitation in establishing the temporal sequence of clonal evolution.

## Conclusions

This case illustrates a rare but serious late complication of CD19 CAR-T cell therapy, characterized by an exceptionally long 24-month latency and documented evolution from wild-type TP53 to biallelic loss. While causality cannot be established from a single case, these findings are consistent with the hypothesis that prolonged genotoxic stress and inflammatory cytokine exposure may promote clonal selection of TP53-mutated hematopoietic cells. This report underscores the necessity of extended hematologic surveillance beyond the first 12 months post-CAR-T infusion, baseline CHIP screening prior to therapy, and systematic longitudinal genomic profiling in future cases to validate these observations.

### Recommendations

Given the practical limitations identified in this report, we recommend that future cases of t-MN following CAR-T therapy include: (i) prospective measurement of CRS-associated cytokines (IL-6, TNF-α, GM-CSF) during and after CAR-T infusion to directly assess inflammation-driven clonal selection; (ii) standardized pre-CAR-T CHIP screening using uniform high-sensitivity NGS panels to establish baseline clonal architecture; and (iii) systematic long-term hematologic follow-up beyond 24 months to define the true duration of t-MN risk.

## Data Availability

All clinical raw data, pathological images, karyotype figures, PET/CT scans and clinical timeline records generated and analyzed during this case study are fully included within the article and its figures. No high-throughput sequencing data requiring public repository deposition was produced for this research.
